# The Neuroscience of the Flow State: Involvement of the Locus Coeruleus Norepinephrine System

**DOI:** 10.3389/fpsyg.2021.645498

**Published:** 2021-04-14

**Authors:** Dimitri van der Linden, Mattie Tops, Arnold B. Bakker

**Affiliations:** ^1^Department of Psychology, Education, and Child Studies, Erasmus University Rotterdam, Rotterdam, Netherlands; ^2^Developmental and Educational Psychology Unit, Leiden University, Leiden, Netherlands; ^3^Department of Industrial Psychology and People Management, University of Johannesburg, Johannesburg, South Africa

**Keywords:** flow state, locus coeruleus, norepinephrine, task engagement, peak experience, human performance

## Abstract

Flow is a state of full task engagement that is accompanied with low-levels of self-referential thinking. Flow is considered highly relevant for human performance and well-being and has, therefore, been studied extensively. Yet, the neurocognitive processes of flow remain largely unclear. In the present mini-review we focus on how the brain's locus coeruleus-norepinephrine (LC-NE) system may be involved in a range of behavioral and subjective manifestations of flow. The LC-NE system regulates decisions regarding task engagement vs. disengagement. This is done *via* different modes of baseline and stimulus-evoked norepinephrine release. We emphasize the theoretical and empirical overlap between the LC-NE system and flow. For both, a match between a person's skill and task challenge is important in order to induce high levels task-related attention. Moreover, psychophysiological indicators of LC-NE system activity, such as eye pupil diameter and arousal are also sensitive to flow states. Flow is related to arousal in an inverted U-shape. Similarly, in theories on the LC-NE system, task engagement is highest with intermediate levels of arousal. We argue that knowledge about the role of the LC-NE system in establishing the flow experience may help to gain fundamental knowledge of flow and can contribute to unifying various empirical findings on this topic.

## Introduction

A well-known phenomenon in research on human performance is “flow” (Csikszentmihalyi, 1990, 2014), a state of full task engagement and low levels of self-referential thinking (e.g., worrying, self-reflection). Flow is often associated with athletes, artists, or scientists who are fully task-absorbed in order to achieve peak performance. Yet, flow-like states also occur in more mundane situations, such as when engaging in certain tasks during work or leisure time (Bakker, 2008; Demerouti et al., 2012; Csikszentmihalyi, 2014). An example is a gamer spending hours behind the computer without feeling bored, fatigued, or hungry. Experiencing flow is accompanied with sense of accomplishment, meaningfulness, and positive mood states (Csikszentmihalyi and Nakamura, 2010), and as such, flow also plays a role in well-being.

Flow has been extensively studied in the past decades (Bruya, 2010; Csikszentmihalyi, 2014; Harmat et al., 2016), but relatively few studies have focused on its neurocognitive basis. This is unfortunate, because insight in the fundamental processes of flow would allow the needed interdisciplinary and systematic scrutiny of the topic that goes beyond self-reports and behavioral observations. Also, knowledge about the brain processes could help to examine whether flow has unique features, or alternatively, may simply reflect an extreme level of task focus or sustained attention (Unsworth and Robison, 2017). In case of the latter, flow possibly may have to be assimilated in the general attentional literature.

Although neuroscientific research on flow is limited, already in 2004, Dietrich suggested that during flow, the frontal lobes may be less active, indicating that much of the behavioral regulation is bottom-up (i.e., automatic). In addition, Ulrich et al. (2014, 2016) used functional magnetic resonance imaging (fMRI), to examine the various brains areas are active or inactive during flow. They could not confirm the hypofrontality account of flow (Dietrich, 2004) because dorsolateral prefrontal areas were quite active during flow. However, frontal areas related to self-reflective thinking were less active. Using the source localization functions of electroencephalogram (EEG), Leroy and Cheron (2020) followed the brain activity of a professional tightrope performer. In line with hypofrontality theory, periods of flow were characterized by lowered frontal lobe activity, compared to more stressful task periods. Besides such specific neuropsychological findings there also has been increased effort to integrate such findings into more comprehensive models on how the brain establishes flow (e.g., Harris et al., 2017). Van der Linden et al. (2021) proposed a neuroscientific model on how flow relates to functional brain networks.

The present review focuses on the presumed role of the *locus coeruleus norepinephrine* (LC-NE) system in flow. The locus coeruleus is a small nucleus in the pons that is responsible for most of the norepinephrine release in the brain (Benarroch, 2009). To the best of our knowledge, the first time the possible relationship between the LC-NE system and flow was proposed, was in the review of Van der Linden et al. (2021). Nevertheless, it was described comparatively briefly. In the present review, we will elaborate on this relationship. Understanding the role of the LC-NE system in flow is imperative because, as we will argue, it would allow a reconciliation of the literature on arousal and the neuroscience of attention, with the literature of flow.

## Basic Dimensions of Flow

It has been established that, in order to experience flow, a key dimension is the match between a person's skills and the task challenges (Keller, 2016). A too easy task more likely leads to boredom, rather than flow. A too difficult task often leads to frustration, stress or lack of interest, which are all states that are largely incompatible with flow (Bakker and Oerlemans, 2011). Such skill-challenge match, that is central to flow, already hints at a possible involvement of the LC-NE system (see below).

Another defining flow characteristic is the strong attentional focus, sometimes referred to as task engagement or absorption (Martin and Jackson, 2008). This implies the inhibition of task-irrelevant stimuli or thoughts. The brain's central executive network (CEN) is presumed to play a relevant role in this flow-related focus (e.g., Harris et al., 2017; Van der Linden et al., 2021). The CEN is a collection of brain areas that support higher-order cognitive functions such as working memory, attention, and inhibition (Bressler and Menon, 2010).

Low levels of self-referential thinking are a third hallmark of flow (Nakamura and Csikszentmihalyi, 2014). During flow, stress levels are low and so are worries and self-reflective thinking. The presumed brain network associated with this is the *Default Mode Network* (Van der Linden et al., 2021), which is typically active when *not* engaging in an external cognitive task (Bressler and Menon, 2010). Brain imagining studies have confirmed that activity of the default mode network is indeed lowered during flow states (Ulrich et al., 2014, 2016).

The literature also provides a list of feelings and perceptions involved in flow. People who experienced flow often, at least retrospectively, reported feeling in control, having a clear sense of direction (i.e., clear goals), and a condensed perception of time (Csikszentmihalyi, 2014). The latter means that time seems to fly when people are in a flow (Hancock et al., 2019). In their review, Van der Linden et al. (2021) speculated that such flow-related changes in time perception may be linked to the reduced sense of self, mediated by parts of the insular cortex.

## Flow and Motivational Brain Systems

In lab studies, several large-scale brain systems have already been studied in relation to flow. One finding is that areas related to the brain's dopaminergic reward system are more active during flow (Ulrich et al., 2014, 2016). Activity of the reward system tends to coincide with feelings of optimism and hope, positive mood, and feeling energized or motivated (Ashby et al., 1999). In addition, dopamine can reduce feelings of fatigue or discomfort (e.g., coffee indirectly increases dopamine: Lorist and Tops, 2003). These properties of the dopaminergic reward system, thus, fit with important dimensions of flow, such as intrinsic motivation, and a relentless dedication to a task.

In comparison to dopaminergic systems in flow, less attention has been given to the LC-NE system. The possible role of the LC-NE system in flow was proposed for the first time, albeit relatively briefly, in a previous review (Van der Linden et al., 2021). However, that review did not mention the link with pupil measure studies, and did not refer to connections between the LC-NE system and effort and the DMN in relation to flow. Therefore, the present short review contributes by discussing a wider range of empirical findings supporting the involvement of the LC-NE system in flow.

## Basic Characteristics of the LC-NE System

The locus coeruleus is largely responsible for releasing central NE, and has widespread afferent connections to areas such as the cerebral cortex, cerebellum, hippocampus, and the ventral tegmental area (Benarroch, 2009). As such, it has a broad influence on the brain's general state and interacts with many other brain systems. Initially, it was assumed that the LC-NE system was mainly responsible for the brain's level of arousal (Berridge and Waterhouse, 2003), but in their seminal paper, Aston-Jones and Cohen (2005), pointed out that it has more complex functions. They proposed that, basically, the LC-NE regulates decisions on task engagement vs. disengagement, based on trade-offs between task rewards vs. its costs. If the tradeoff favors rewards, then the LC-NE system facilitates a brain state supporting task-relevant information processing, while simultaneously neglecting or actively suppressing task irrelevant stimuli. This brain state manifests itself as a high task engagement, also referred to as task *exploitation* (Aston-Jones et al., 2000). High engagement/exploitation involves the investment of time and effort in order to reap current or expected benefits of the task.

If, however, the costs will outweigh the benefits, then LC-NE system activity changes such that it becomes more difficult to uphold task engagement and there will be a tendency to get distracted or enter an “off-focus state” (Mittner et al., 2016). This latter state has been described as *exploration* (Aston-Jones et al., 2000), because the brain is then searching for alternative activities or stimuli that may be more rewarding than the current ones (Aston-Jones and Cohen, 2005). In light of this, the LC-NE system can be said to play a role in “decisions” on exploitation, -should I continue to put effort into the task at hand?- vs. exploration, -are there better options for me to engage in?

**Figure 1:**
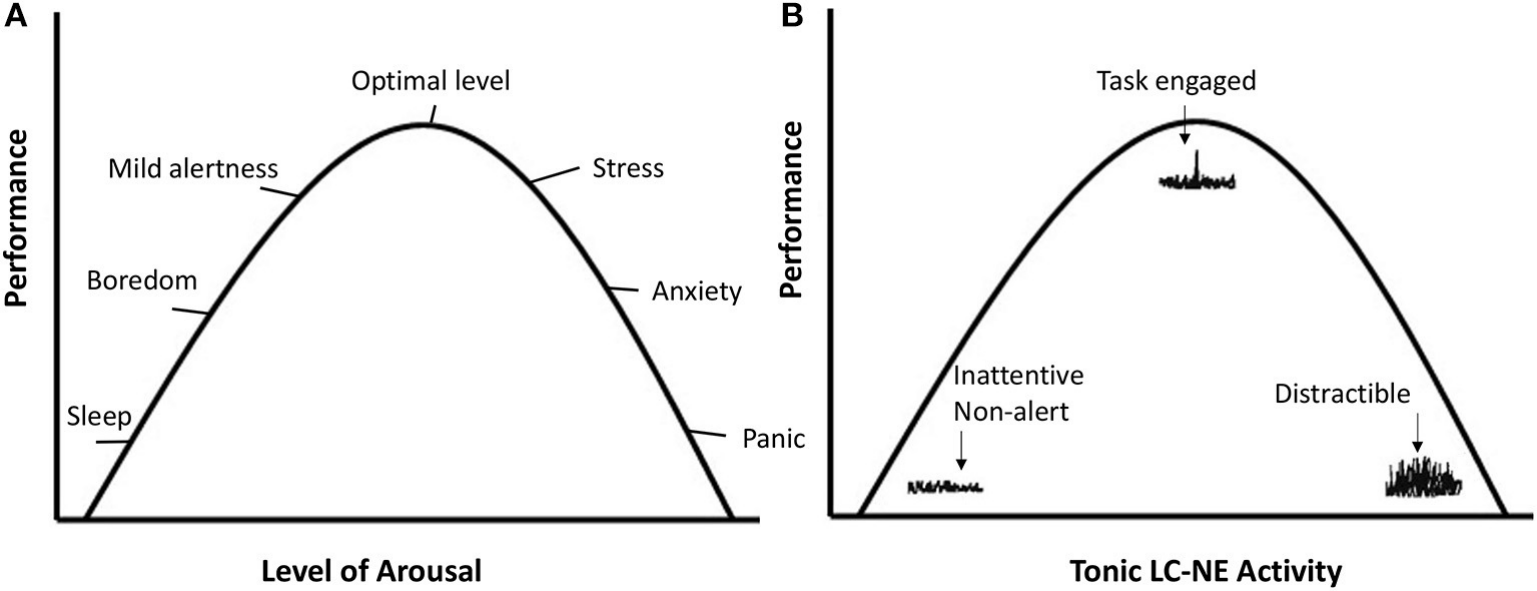
**(A)** Reflects the mood states and performance as a function of the level of arousal. **(B)** Plots performance as a function of tonic and phasic LC-NE activity in line with the theory of Aston-Jones and Cohen (2005). On the left side, tonic LC-NE is low and so are phasic responses to stimuli. This has been referred to as the disengagement mode (Hopstaken et al., 2015). With intermediate tonic LC-NE activity, phasic LC-NE responses are strong to task-relevant stimuli. This is the exploitation mode, associated with optimal engagement and performance. On the right side, tonic LC-NE is high, and phasic LC-NE responses are undifferentiated. This is the exploration mode.

The LC-NE system regulates exploitation vs. exploration through patterns of phasic and tonic NE release. *Phasic* refers to short bursts of NE as a reaction to stimuli. *Tonic* refers to the baseline or background level of NE. The different LC-NE output modes are depicted in Figure 1B. With intermediate tonic NE levels, phasic NE reactions to task relevant stimuli tend to be strong, and high task engagement occurs. Hence it is referred to as the *exploitation mode* of the LC-NE system.

When tonic NE is high, phasic responses become less differentiated and respond to a broader range of stimuli, which indicates exploration of the environment, or susceptibility to distraction. Aston-Jones and Cohen (2005) referred to this state as the *exploration mode*. On the left-side of Figure 1B, tonic NE is low and phasic NE responses weak, indicating a general unresponsiveness to stimuli. More recently, Hopstaken et al. (2015) referred to this as the *disengagement mode*, which is associated with feelings of fatigue and boredom.

Highly relevant for the present review is that presumed indicators of LC-NE activity have been confirmed to respond to reward-cost trade-offs. Illustrative are the pupilometry studies, because pupil dilation is assumed to partly reflect tonic and phasic LC-NE activity (Gilzenrat et al., 2010; Jepma and Nieuwenhuis, 2011; Elman et al., 2017). Gilzenrat et al. (2010) conducted a study in which participants could earn money by correct responses to task trials. Over time, the trials became more difficult and, thus required more effort, but more money could be gained. Participants had access to a reset button by which they could set trial-difficulty level back to baseline, although the money earned per trial decreased accordingly then. In those studies, baseline (tonic) pupil diameter and (phasic) task-related pupil responses both increased with increasing trial difficulty and reward, up to the point at which participants were maximally engaged. At some point, however, the trials became so difficult that success was unlikely. In that case, the costs (e.g., effort) exceeded the potential rewards, and participants often pressed the reset button. A key finding was that the time participants pressed the reset button could be predicted by their pattern of high tonic pupil diameter and weaker and undifferentiated phasic pupil responses. In other words, the changing trade-off between rewards and effort seemingly shifted the participants from an exploitation to an exploration mode.

## Flow State and the LC-NE System

The link between the LC-NE exploitation mode and task engagement, as depicted in Figure 1, already suggests that the highly focused task behavior that is prototypical of flow, may not be possible without the proper LC-NE configuration. When the LC-NE system is in the alternative disengagement or exploration mode, this is accompanied with feelings of boredom/fatigue/inattentiveness or frustration/stress/distraction, respectively. Those feelings have been shown to be largely incompatible with flow (see Bakker and Oerlemans, 2011, for a theoretical analysis).

Flow as well as the LC-NE exploitation mode both strongly depend on a match between skill level and task challenge (e.g., difficulty). To illustrate, in laboratory studies on flow, researchers usually compare different conditions (Ulrich et al., 2014, 2016; Tozman and Peifer, 2016; Katahira et al., 2018). This often involves an *underload* or *boredom condition* in which the task is relatively easy, and an *overload* or *stress/frustration condition*, in which the task is too difficult. These conditions are then compared to a *flow condition* in which the task difficulty is matched with the participant's skill level.

Compared to the underload and overload conditions, participants indeed show the most behavioral and subjective flow indications in the flow condition (Ulrich et al., 2014, 2016; Tozman and Peifer, 2016; Katahira et al., 2018). One key insight is that the three conditions outlined above map well onto the three modes of the LC-NE system: Disengagement (similar to the boredom condition), exploitation (flow), and exploration (overload). See also Figure 1A.

In the previous section, we referred to the study of Gilzenrat et al. (2010) in which participants shifted from an exploitation to exploration mode when the effort they had to invest in a trial (due to trial difficulty) did no longer match the expected reward. In flow research, similar effects seem to occur. Intrinsically motivated people initially tend to exert *more* effort and experience *more* flow-like symptoms when a task becomes more challenging (Csikszentmihalyi and Nakamura, 2010). However, when the required effort does not lead to the desired outcome, -e.g., if errors increase, no matter how hard one tries-, the cost-reward tradeoff becomes unfavorable and flow gets disrupted (Keller, 2016). In that case, self-referential thinking (e.g., worries, stress, self-reflection) and distractibility increase. The latter indicates a shift toward the exploration mode.

In this process, the literature on the link between the LC-NE system on the one hand, and the DMN and effort, on the other hand, may be relevant too. Specifically, Mittner et al. (2016) explained how fluctuations in LC-NE output are closely intertwined with DMN activity in relation to “off-task states” and self-referential thinking. They stated that DMN activity is down-regulated by the exploitation mode, and up-regulated by the exploration mode (see also Ross and Van Bockstaele, 2021). This suggest that the LC-NE system may play an even more central role in flow than was indicated in Van der Linden et al. (2021), because the system would not only relate to attentional focus, but may also contribute to lowered levels of self-referential thinking during flow.

With regard to effort, Borderies et al. (2020) found that, in rhesus monkeys, reductions in LC-NE levels–through clonidine- were related to lowered motivation to work for rewards. Mainly the effort-based decisions were influenced by LC-NE, whereas the processing of rewards remained unaffected. In an exploitation mode, LC-NE facilitated effort expenditure, whereas in an exploitation or disengagement mode, task-related effort was reduced. Those findings are in line with the notion that being in an exploitation mode during flow, is typically accompanied with the motivation and ability to work on a task relentlessly.

The present review describes theoretical and empirical findings in accordance with the proposition that the LC-NE system is involved in typical flow characteristics. Yet, one limitation is that there are currently no published articles that have *explicitly* tested the link between the LC-NE system and flow. This, however, does not mean that there is no empirical evidence in that direction. Specifically, based on a range of studies, Peifer et al. (e.g., 2014) concluded that arousal shows a reversed U shape pattern with regard to flow. Too low or too high arousal levels are associated with boredom/fatigue and frustration/stress, respectively. Flow requires an intermediate level of arousal that Peifer et al. (2014) described as “optimized physiological activation.” Given that the LC-NE system plays a pivotal role in the brain's arousal level (Berridge and Waterhouse, 2003; Aston-Jones and Cohen, 2005), these findings are clearly in line with the flow-LC-NE hypothesis we emphasize here.

Additional evidence comes from gaming and digital media research, using pupilometry to assess flow states. Mauri et al. (2011) found that, participants who had to do various tasks on Facebook had higher mean pupil dilation in an overload condition than in a flow condition. This fits with the flow-LC-NE hypothesis because mean pupil size has been linked to tonic NE levels (Gilzenrat et al., 2010). Not in line with this hypothesis was that they also found higher mean pupil size in a relaxation condition. On the other hand, it seems plausible that in the relaxation condition of Mauri et al.'s study, participants were not necessarily under-aroused or bored, but may have been open to environmental input (i.e., in an exploration mode), hence their higher baseline pupil diameter. Also, although LC-NE activity and pupil responses are correlated, they may differ during various task epochs (Yang et al., 2020).

## Concluding Remarks and Future Research

The current mini-review went beyond previous work in this area (i.e., Van der Linden et al., 2021), by presenting new lines of arguments and evidence regarding the relationship between flow and the LC-NE system. Knowledge about such a relationship is important for several reasons. First, it is able to provide a unifying, fundamental, explanation for why pupil size, arousal, a skill-challenge match, attentional focus, and reduced self-referential thinking are all related to flow. Our current emphasis on the notion that LC-NE is involved in flow, does not negate the involvement of several other neuromodulatory brain systems. For example, there is substantial molecular, cellular, and physiological overlap between DA and NE systems and the LC can simultaneously broadcast both DA and NE across the brain. Ranjbar-Slamloo and Fazlali (2020), suggested that DA and NE may function in parallel to maintain the states required for normal cognitive processes. In addition, Ulrich et al. (2016) argued that, *via* serotoninergic output, the dorsal raphe nucleus downregulates the medial prefrontal cortex (a key structure of the DMN) during flow. Phasic serotonin responses in this nucleus have also been found to influence pupil responses (Cazettes et al., 2021). Therefore, future research may want to try to disentangle the specific influences of the various neuromodulatory systems.

Accordingly, we believe that the insight of LC-NE involvement in flow may, more generally, guide future research in this area. For example, futures studies may want to directly test whether flow is associated with the expected pattern (i.e., exploitation) of pupil diameter responses. Moreover, psychopharmacological interventions may be used to examine the relative contributions of different neuromodulators and their interactions. Combinations of pupilometry and electroencephalogram (EEG) could be used to tests associations with behavioral and subjective indicators of flow, thereby exploring the possibility of non-invasive brain measures of flow states. All in all, we hope that our review will inspire such, and other, studies on the neuroscientific basis of the flow state.

## Author Contributions

DL drafted the first version of the manuscript. MT and AB participated in writing and critical revision of the manuscript. All authors approved the final version.

## Conflict of Interest

The authors declare that the research was conducted in the absence of any commercial or financial relationships that could be construed as a potential conflict of interest.
